# Vaccination Utilization and Subnational Inequities during the COVID-19 Pandemic: An Interrupted Time-Series Analysis of Administrative Data across 12 Low- and Middle-Income Countries

**DOI:** 10.3390/vaccines11091415

**Published:** 2023-08-24

**Authors:** George Mwinnyaa, Michael A. Peters, Gil Shapira, Rachel Neill, Husnia Sadat, Sylvain Yuma, Pierre Akilimali, Shahadat Hossain, Naod Wendrad, Wisdom K. Atiwoto, Anthony Adofo Ofosu, Jean Patrick Alfred, Helen Kiarie, Chea Sanford Wesseh, Chris Isokpunwu, Desmond Maada Kangbai, Abdifatah Ahmed Mohamed, Kadidja Sidibe, Salome’ Drouard, Pablo Amor Fernandez, Viviane Azais, Tawab Hashemi, Peter M. Hansen, Tashrik Ahmed

**Affiliations:** 1 The Global Financing Facility for Women, Children, and Adolescents, Washington, DC 1818, USA; 2 The World Bank, Washington, DC 20433, USAgshapira@worldbank.org (G.S.);; 3 Ministe’re de la Sante, Kinshasa 4310, Democratic Republic of the Congo; 4 Kinshasa School of Public Health, University of Kinshasa, Kinshasa P.O. Box 11850, Democratic Republic of the Congo; 5 Ministry of Health and Family Welfare, Dhaka 1000, Bangladesh; 6 Ministry of Health, Addis-Ababa 1234, Ethiopia; 7 Ministry of Health, Accra P.O. Box M 44, Ghana; 8 Ghana Health Service, Accra P.O. Box M 44, Ghana; 9 Ministère de la Sante Publique et de la Population, Port-au-Prince HT6123, Haiti; 10 Ministry of Health, Nairobi P.O. Box 30016-00100, Kenya; 11 Ministry of Health, Monrovia 20540, Liberia; 12 Federal Ministry of Health, Abuja 900242, Nigeria; 13 Ministry of Health and Sanitation, Freetown 232, Sierra Leone; 14 Federal Ministry of Health & Human Services, Mogadishu 28RX+5W6, Somalia

**Keywords:** health inequalities, immunization, routine data, child health, health systems

## Abstract

Background: During and after the SARS-CoV-2 (COVID-19) pandemic, many countries experienced declines in immunization that have not fully recovered to pre-pandemic levels. This study uses routine health facility immunization data to estimate variability between and within countries in post-pandemic immunization service recovery for BCG, DPT1, and DPT3. Methods: After adjusting for data reporting completeness and outliers, interrupted time series regression was used to estimate the expected immunization service volume for each subnational unit, using an interruption point of March 2020. We assessed and compared the percent deviation of observed immunizations from the expected service volume for March 2020 between and within countries. Results: Six countries experienced significant service volume declines for at least one vaccine as of October 2022. The shortfall in BCG service volume was ~6% (95% CI −1.2%, −9.8%) in Guinea and ~19% (95% CI −16%, 22%) in Liberia. Significant cumulative shortfalls in DPT1 service volume are observed in Afghanistan (−4%, 95% CI −1%, −7%), Ghana (−3%, 95% CI −1%, −5%), Haiti (−7%, 95% CI −1%, −12%), and Kenya (−3%, 95% CI −1%, −4%). Afghanistan has the highest percentage of subnational units reporting a shortfall of 5% or higher in DPT1 service volume (85% in 2021 Q1 and 79% in 2020 Q4), followed by Bangladesh (2020 Q1, 83%), Haiti (80% in 2020 Q2), and Ghana (2022 Q2, 75%). All subnational units in Bangladesh experienced a 5% or higher shortfall in DPT3 service volume in the second quarter of 2020. In Haiti, 80% of the subnational units experienced a 5% or higher reduction in DPT3 service volume in the second quarter of 2020 and the third quarter of 2022. Conclusions: At least one region in every country has a significantly lower-than-expected post-pandemic cumulative volume for at least one of the three vaccines. Subnational monitoring of immunization service volumes using disaggregated routine health facility information data should be conducted routinely to target the limited vaccination resources to subnational units with the highest inequities.

## 1. Introduction

Vaccination is one of the most cost-effective public health interventions with the greatest contribution to health besides clean water and sanitation [1,2]. Childhood vaccinations alone prevent 4 to 5 million deaths each year [3]. As health systems responded to the SARS-CoV-2 (COVID-19) pandemic, many countries experienced declines in immunization, and some health services have not fully returned to pre-pandemic levels [4,5]. In August 2020, the World Health Organization (WHO) released draft guidelines for immunization catch-up activities, encouraging countries to implement policies and interventions to make sure children who missed any immunization dose are identified and vaccinated [6]. Understanding national and subnational variations in the recovery of immunization services since the beginning of the COVID-19 pandemic is essential for targeting catch-up efforts.

The declines in vaccination during the COVID-19 pandemic occurred within a broader context of persistent immunization coverage inequities, making it especially critical to identify subnational variations in service volume recovery. The 2019 WHO and UNICEF annual national immunization coverage (WUENIC) report estimated that 85% of infants received the three doses of the DPT3 vaccine, leaving 20 million children under- or unimmunized worldwide [7]. Globally, the coverage of DPT3 and measles has remained constant at 85% since 2010 [7]. The challenges of immunizing “hard-to-reach” populations in remote rural areas, informal settlements, and conflict-impacted areas are well documented [8,9,10,11]. Immunization programs in most low- and middle-income countries (LMICs) face considerable challenges in reaching their most vulnerable children, including (1) parent/caretaker related factors (vaccination knowledge [9,12], misconception [10,12,13,14], lack of trust [14,15,16], and financial challenges [13]); and (2) health system challenges (inadequate cold chain [11], vaccine supply, and distribution challenges [17], human resource [13] and infrastructure barriers [17], the distance between facilities and populations [18]), and (3) provider related barriers (attitude towards parents/caregivers [19,20]).

The COVID-19 pandemic compounded these challenges, leading to substantial immunization coverage declines. Demand for immunization services was reduced due to movement restrictions, and parents were unwilling to risk exposure to bring their children to health facilities [21,22,23,24]. On the supply side, health facility staff responsible for routine immunization services were reassigned to COVID-19 response activities, finances and equipment for routine immunization were diverted to the COVID-19 response, supply chain and logistics challenges led to shortages of routine vaccines, and the closure of facilities and immunization outreach services was common [25,26,27].

These challenges, and the subsequent recovery in immunization service volumes, have been uneven, compounding existing inequities in immunization coverage. Turning the tide on immunization coverage declines requires access to readily available data to track and identify subnational units that are experiencing disruptions in immunization service volumes in real time. Luckily, such data exist in the form of routine health information management systems (HMIS) data.

Health facilities collect and report monthly immunization service volume data from the point of service delivery into administrative data systems, enabling timely monitoring of immunization services at the subnational level. While global coverage estimates are important for alignment, they do not provide real-time indications of when and where gaps in child immunization services are emerging. HMIS-based data collection and analysis can help Ministries of Health and their partners rapidly identify and provide targeted responses to subnational declines in immunization service volumes.

HMIS data usage is often hampered by data quality challenges, including incomplete reporting and data consistency challenges [28,29,30,31]. However, it remains the timeliest and most cost-effective data source for monitoring immunization programs, particularly at the subnational level. Documented routine health facility data quality assessments and transparent adjustment methods can mitigate data quality challenges and produce plausible trends, disruptions, and coverage estimates using these data [29,32].

This study uses health facility-reported service volume data to estimate the variability between and within countries in immunization service recovery for three priority indicators (BCG, DPT1, and DPT3) since the onset of the COVID-19 pandemic. The objective of this analysis is to identify countries and subnational units that may require additional resources and interventions to recover from ongoing service delivery challenges and improve their immunization service volumes to pre-pandemic levels.

## 2. Materials and Methods

The Global Financing Facility for Women, Children, and Adolescents (GFF) partnered with countries during the COVID-19 pandemic to support the monitoring of essential reproductive, maternal, and child health services. This monitoring exercise assessed the trends in service volume data reported by health facilities in each country using national HMIS data. These data have been available from before the onset of the pandemic to the present. Therefore, the trends projected from pre-pandemic service volumes provide a counterfactual—what the service volume should have been had the pandemic not occurred.

This analysis focuses on routine child immunization services in 12 countries: Afghanistan, Bangladesh, the Democratic Republic of Congo (DRC), Ethiopia, Ghana, Guinea, Haiti, Kenya, Liberia, Nigeria, Sierra Leone, and Somalia. All analyses were stratified at the subnational unit, representing the first level of administrative hierarchy (e.g., region or province), except in Bangladesh where the second administrative unit was used for the analysis. Data were downloaded in January 2023 for each month between January 2018 and October 2022. The dataset is a census dataset representing all facilities reporting to the HMIS in each country. In Somalia, the dataset does not include Somaliland, which maintains a separate accounting. The Bangladesh data represent only higher-level facilities (excluding community clinics). The dataset also biases facility-based vaccinations because most countries do not include data on vaccines administered during vaccination campaigns in their HMIS reporting.

The analysis is focused on three vaccinations: DPT1, BCG, and DPT3. Data on these three vaccinations are consistently available, are of a high quality across LMICs, and are proxies for other childhood vaccinations which occur at a similar point in the recommended immunization schedule [4]. Further, service coverage of DPT3 is shown to be a good measure of the access, utilization, and performance of the immunization system at the national and sub-national levels [33,34].

### 2.1. Data Quality Assessment and Adjustment

Administrative data are self-reported by health facilities and pose several inferential challenges. The chief source of bias is representativeness; most countries do not have legally mandated reporting by private facilities, and thus the available data primarily reflect the public sector. This challenge is less concerning for child immunization services because they are predominantly administered within the public sector in the sampled countries. Still, in countries where private sector administration is non-negligible, there are limited pathways that would result in differential utilization. Many COVID-19 pandemic constraints, such as disruptions to the global supply chain, movement restrictions, and changes in the demand for health services, would affect all sectors.

Details on the quality assessment and adjustment procedures are described in detail in the previous literature [4]. In brief, we assessed facility-level reporting completeness and identified and adjusted the data for outlier values. We define completeness as the proportion of health facilities that report in a given month out of all facilities expected to report during the given year.

Outliers were defined using two methods. The first definition defines outliers as observations of more than 10 deviations above the positive median absolute deviation (MAD) of the service volume for that same facility during the analysis period. The threshold of 10 was conservative, aimed at identifying egregious data errors but less likely to overcorrect for facilities with regular fluctuations in service volumes. This rule only applied when the identified outlier was above a minimum volume of 100 to avoid over-correcting among low-volume facilities. The second definition of outliers was monthly service volumes which contributed more than 80% of the service volume for the entire year. Outliers identified through either of the two definitions were removed from the analysis. The outlier rate for each country was low, often below 1%, with no consistent change from before the pandemic compared to after the pandemic.

### 2.2. Estimation of Immunization Service Utilization Disruptions and Recovery

The quality-adjusted data were then used to estimate the impact of COVID-19 on immunization service utilization. Interrupted time series regression analysis estimated the expected service volume for each immunization service and subnational unit, assuming the COVID-19 pandemic did not occur. The point of interruption was defined as March 2020, when WHO issued the pandemic declaration. March 2020 also corresponded to the start of global and local pandemic-related restrictions, including travel bans, public gathering restrictions, and lockdowns in most countries [35]. The following linear regression model was used to estimate the expected service volume in each subnational unit in the absence of COVID-19.

$$Y_{tfx}=\beta_{0}+\beta_{1}T+\beta_{2\ldots 12}Month+\beta_{13\ldots 29}{PandemicMonth}_{t}+\alpha_{f}+\varepsilon_{tf}$$

where $Y_{tfx}$ represents the immunization service volume for antigen x, reported by facility f in month t. T is the time in months since January 2018 to account for secular trends ($\beta_{1}$), Month refers to calendar months to account for seasonality ($\beta_{2\ldots 12}$), and $\alpha_{f}$ is the facility-level fixed effect to adjust for time-invariant facility-level factors. PandemicMonth denotes a series of dummy variables for each month between March 2020 and October 2022. While seasonality is expected to persist, we do not make the same assumptions about secular trends. Hence the trend-adjusted change in service volume for each month since the pandemic is defined as $\beta_{13\ldots 29}{PandemicMonth}_{t}-\beta_{1}T$. We report this as the percent deviation from the expected volume by dividing the volume change by the observed service volume. All analyses were carried out using Stata [36], and the visuals were produce using Tableau [37].

## 3. Results

The sample characteristics are shown in Table 1. Across the 12 countries, a total of 79,116 facilities from 303 subnational areas were included in the analysis. The total number of facilities per country ranged from 447 in Guinea to 23,561 in Nigeria. Reporting completeness varied between countries over time. In 2018 and 2019, the reporting completeness for BCG ranged from 67% in DRC to 100% in Bangladesh. In 2020, 2021, and 2022, the reporting completeness for BCG ranged from 50% in DRC to 99% in Bangladesh. The reporting completeness was consistent within countries over time, except for BCG reporting. The yearly volatility in reporting was highest for BCG vaccinations. In DRC, the reporting completeness for BCG ranged from 50% to 73%. A similar year-to-year volatility was observed in Haiti. The completeness for DPT1 and DPT3 was ~80% or higher for almost all countries.

**Figure 1:**
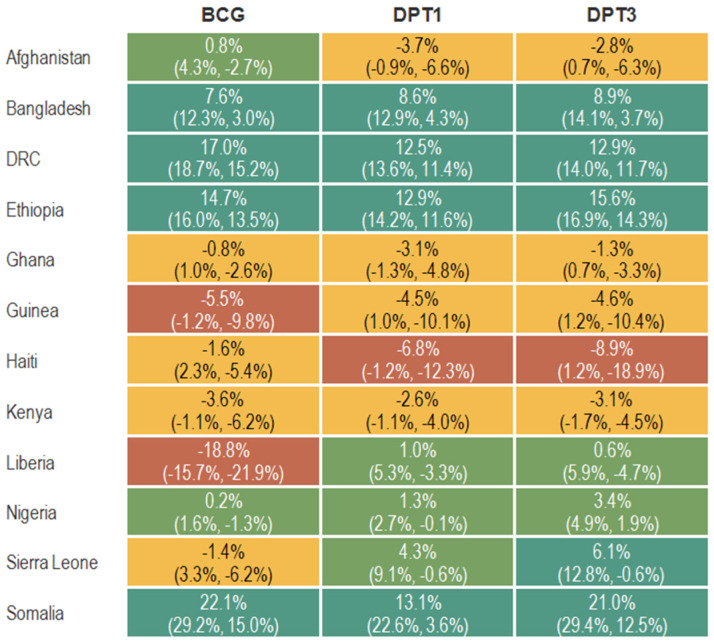
Percent change in immunization service volumes from expected levels based on pre-pandemic trends in 12 countries for BCG, DPT1, and DPT33 from March 2020 to October 2022 (β, 95% CI).

The national-level volume change for the BCG, DPT1, and DPT3 vaccinations between the onset of the COVID-19 pandemic in March 2020 and October 2022 is shown in Figure 1. Six countries (Afghanistan, Ghana, Guinea, Haiti, Kenya, Sierra Leone, and Liberia) experienced significant declines in service volume for at least one vaccine. In Guinea, Kenya, and Liberia, there were significant cumulative declines in BCG services volume from March 2020 to October 2022 when compared to pre-pandemic reported service volumes. For example, the shortfall in BCG service volume was ~6% (95% CI −1%, −10%) in Guinea and ~19% (95% CI −16%, 22%) in Liberia. Significant cumulative shortfalls in DPT1 service volume are observed in Afghanistan (−4%, 95% CI −1%, −7%), Ghana (−3%, 95% CI −1%, −5%), Haiti (−7%, 95% CI −1%, −12%), and Kenya (−3%, 95% CI −1%, −4%). Kenya is the only country with a statistically significant shortfall in all three vaccination services. The largest single decline is observed in Liberia for BCG vaccination, followed by Haiti for the DPT vaccination series. In contrast, statistically significant increases in service volume since the baseline period are observed in Bangladesh, DRC, Ethiopia, and Somalia for all three antigens.

**Figure 2:**
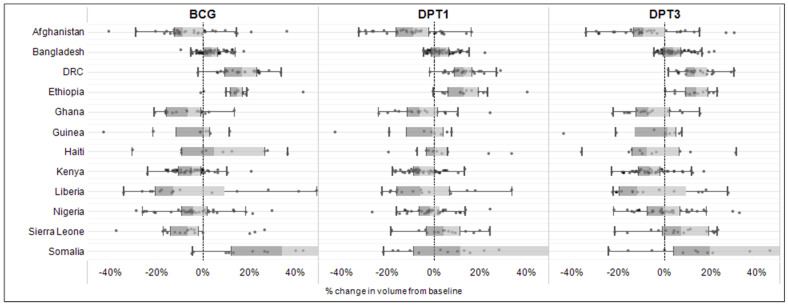
Percent change in immunization service volumes from expected levels based on pre-pandemic trends in 12 countries for BCG, DPT1, and DPT3, by subnational units, from March 2020 to October 2022.

**Figure 3:**
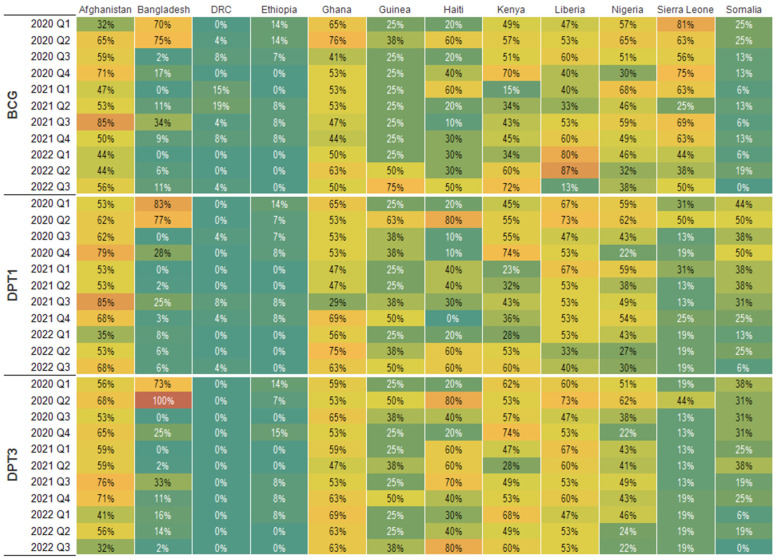
Percent of subnational units with more than 5% shortfall for BCG, DPT1, and DPT3 in 12 countries from March 2020 to October 2022.

Figure 2 shows the results of the subnationally stratified model. The immunization shortfalls at the subnational level demonstrate significant variability within and between countries. In Afghanistan, Ghana, Guinea, Kenya, Liberia, Nigeria, and Sierra Leone, more than half of the subnational units experienced a shortfall in BCG, DPT1, or DPT3 service volume. There are noticeable extreme subnational shortfalls (>30%) in service volume for at least one antigen in Afghanistan, Guinea, Haiti, Liberia, and Sierra Leone. In Figure 3, these subnational area estimates are summarized by quarter and by year. DRC and Ethiopia have the lowest percentage of subnational units reporting a 5% or higher decline in immunization services. In 2020, Sierra Leone had the highest percentage of subnational units experiencing a 5% or higher shortfall in BCG service volume (2020 Quarter 1 (Q1), 81%), followed by Ghana (2020 Q2, 76%) and Bangladesh (2020 Q2, 75%). In 2021 and 2022, Liberia had the highest percentage of subnational units reporting a 5% or higher reduction in BCG service volume (2022 Q2, 87%), followed by Afghanistan (2021 Q3, 85%), and Guinea (2022 Q3, 75%).

Afghanistan had the highest percentage of subnational units reporting a shortfall of 5% or higher in DPT1 service volume (85% in 2021 Q1 and 79% in 2020 Q4), followed by Bangladesh (2020 Q1, 83%), Haiti (80% in 2020 Q2), and Ghana (2022 Q2, 75%). All subnational units in Bangladesh experienced a 5% or higher shortfall in DPT3 service volume in the second quarter of 2020. In Haiti, 80% of the subnational units experienced a 5% or higher reduction in DPT3 service volume in the second quarter of 2020 and the third quarter of 2022.

Between the first and third quarter of 2022, the percentage of subnational units reporting a 5% or higher decline in BCG vaccination service volume increased significantly (>10%) in Afghanistan (from 44% of subnational units to 56%), Guinea (25% to 75%), Haiti (30% to 50%), and Kenya (34% to 72%). The percentage of subnational units reporting 5% or higher declines in BCG service volume decreased in Nigeria (46% to 38%) and Liberia (80% to 13%). Similarly, the percentage of subnational units reporting a 5% or higher decline in BCG vaccination service volume increased significantly between the first and third quarter of 2022 (>10%) in Afghanistan (35% to 68%), Guinea (25% to 50%), Haiti (20% to 60%), and Kenya (28% to 60%). The percentage of subnational units reporting 5% or higher declines in BCG service volume decreased in Nigeria (43% to 30) and Liberia (53% to 40%). Unlike BCG and DPT1 service volumes, the percentage of subnational units reporting a 5% or higher decline in DPT3 vaccination service volume increased between the first and third quarter of 2022 in Guinea (25% to 38%), Haiti (30% to 80%), and Liberia (47% to 53%). The percentage of subnational units reporting 5% or higher declines in DPT3 service volume either decreased or did not change in the other countries.

## 4. Discussion

We identified a substantial variation in service volume changes for BCG, DPT1, and DPT3 vaccinations both between and within countries when compared to the average vaccination service volume observed during the pre-pandemic period. At least one subnational unit in every country had a significantly lower-than-expected cumulative vaccination service volume for at least one of the three vaccines as of October 2022. This finding highlights the subnational inequities in the recovery of routine immunization services and emphasizes the critical importance of subnational estimates to guide recovery efforts.

Focusing only on national immunization service volume or coverage estimates can misinform or misguide global immunization efforts. Our analysis shows that national-level immunization coverage or service volume estimates can mask important subnational inequalities in immunization service delivery. For instance, when considering only national-level immunization shortfalls, Bangladesh, Ethiopia, and DRC would not be eligible for global-level immunization service recovery support programs; however, we identified significant subnational shortfalls which require urgent resources and interventions.

A failure to earmark such countries for global support would result in chronic inequality in immunization service delivery which might go unnoticed by the current global and national-level focused immunization monitoring programs [38,39,40,41]. As volatile subregions become the center of attention for monitoring immunization programs, disaggregating results by subnational areas must become standard practice. Our national-level results align with previously published studies on immunization service disruption and recovery trends during the COVID-19 pandemic [42,43,44,45]. However, our study, unlike other studies [42,44], further demonstrates that the national immunization trends do not necessarily mirror the subnational trends. This phenomenon may similarly explain why countries with high administrative MCV1 coverage estimates experience high measles outbreaks in some subnational units [46,47,48,49,50]. While numerous studies have centered on the effects of COVID-19 on routine childhood vaccination [50], there is limited information on geographic disparities in immunization service delivery [51], especially at the subnational level. Investigating inequities in immunization services is critical to guide health policy and prevent regression in the progress made toward achieving equitable vaccination coverage.

Identifying subnational differences in immunization service shortfalls has immediate practical implications for planning, resource allocation, and the implementation of immunization service volume recovery programs. For example, countries with a high percentage (>60%) of subnational units experiencing declines in immunization service volume (e.g., Afghanistan, Ghana, Haiti, and Kenya) need broader, national-level investments aimed at improving the overall immunization service delivery system. In such countries, short-term catch-up interventions such as mass national immunization campaigns are likely to capture more un-immunized children than children who are already vaccinated. In contrast, countries with a low percentage of subnational units reporting a higher percentage of immunization service volume shortfalls (e.g., Bangladesh, Somalia, and Sierra Leone) can implement more targeted and cost-efficient immunization campaigns targeting selected subnational units with significant shortfalls in their immunization service. 

The findings from this analysis also demonstrate the added value of using disaggregated HMIS-based estimates to monitor immunization programs. The subnational monitoring approach used here can both identify inequities and support the strategic allocation of resources, planning, and the implementation of targeted immunization catch-up activities to reduce immunization service delivery inequities both during recovery and on an ongoing basis. While household surveys will continue to be the gold standard in assessing vaccination coverage, routine health facility data are better placed to capture the effects of shocks and immunization service recovery dynamics. Though data quality concerns often discourage use, the ability of facility-reported programmatic data to establish pre-shock counterfactuals within subnational areas is unmatched. Further, this specific type of counterfactual analysis is robust to the most concerning data quality issues, such as low completeness, if the quality is stable throughout the analysis.

Our study has several important limitations. The volatility in BCG reporting in DRC and Haiti may impact our results because our analysis does not capture zero reports; hence, we may have underestimated the potential vaccine declines in these countries. This study does not estimate coverage or change in vaccination coverage, as the estimates do not account for a change in the number of children of vaccination age. Because population growth is positive in all countries in this analysis, we expect that our estimates of service volume change would be larger than the estimates of coverage change during this same period. This study only captures routine immunization data from public health facilities and does not include data from private health facilities or immunization campaigns. However, childhood vaccinations are provided mostly through routine services by public health facilities [52,53]. Hence failure to capture data from the private sector and community-based campaigns would not contribute to the estimated change in volume in our analysis. Further, acute decreases in completeness often coincide with shocks, which may affect the measurement of service disruptions and recovery. Finally, we chose a small subset of immunization indicators. The disruptions of these three vaccines may not represent the challenges associated with the supply chain, administration, and uptake of other vaccinations.

## 5. Conclusions

Achieving universal health coverage and the health-related Sustainable Development Goals will require the timely monitoring of immunization programs at the subnational level. Given the limited availability of resources and the costs and logistics needed to conduct reliable household surveys, routine health facility data within the existing health systems are the most cost effective and timely data for monitoring immunization programs. The timely identification of gaps and inequities in immunization programs will support global, national, and subnational immunization officers to quickly identify gaps, allocate resources, and implement targeted immunization programs in the pursuit of protecting children from vaccine-preventable diseases.

## Figures and Tables

**Table 1:** Number of sampled subnational units, facilities, and their facility data completeness by year and by vaccine.

			BCG					Penta1					Penta3				
Country	Subnat. Areas	# of Facilities	2018	2019	2020	2021	2022	2018	2019	2020	2021	2022	2018	2019	2020	2021	2022
Afghanistan	34	2601	86%	85%	87%	84%	88%	87%	86%	87%	84%	87%	86%	86%	87%	84%	87%
Bangladesh	64	654	100%	99%	96%	98%	99%	100%	100%	98%	97%	98%	100%	100%	97%	98%	98%
DRC	26	11,253	72%	67%	72%	50%	73%	79%	83%	87%	79%	88%	79%	83%	87%	79%	88%
Ethiopia	14	22,618	74%	80%	80%	80%	80%	83%	91%	91%	89%	88%	83%	91%	91%	88%	88%
Ghana	16	7803	79%	77%	77%	77%	76%	92%	90%	91%	90%	89%	93%	92%	93%	92%	91%
Guinea	8	447	91%	95%	97%	98%	78%	92%	95%	98%	99%	95%	91%	95%	97%	98%	94%
Haiti	10	636	79%	70%	70%	64%	75%	87%	83%	84%	84%	85%	86%	81%	82%	83%	84%
Kenya	47	7047	81%	79%	77%	77%	79%	88%	88%	87%	86%	89%	88%	88%	87%	86%	89%
Liberia	15	665	94%	94%	94%	90%	94%	95%	94%	93%	90%	94%	95%	94%	93%	90%	94%
Nigeria	37	23,561	77%	83%	81%	79%	81%	83%	88%	84%	81%	83%	83%	88%	84%	81%	83%
Sierra Leone	16	1347	97%	97%	95%	96%	93%	98%	98%	95%	98%	94%	98%	98%	95%	98%	94%
Somalia	16	485	74%	78%	82%	85%	78%	78%	82%	85%	86%	77%	77%	82%	84%	86%	76%

# means “number”.

## Data Availability

The data underlying this article were provided by and are the property of the ministries of health of the 12 countries participating in the analysis. The data will be shared on reasonable request with the permission of the 12 ministries from gffsecretariat@worldbank.org.
